# Deletion in the Bardet–Biedl Syndrome Gene *TTC8* Results in a Syndromic Retinal Degeneration in Dogs

**DOI:** 10.3390/genes11091090

**Published:** 2020-09-18

**Authors:** Suvi Mäkeläinen, Minas Hellsand, Anna Darlene van der Heiden, Elina Andersson, Elina Thorsson, Bodil S. Holst, Jens Häggström, Ingrid Ljungvall, Cathryn Mellersh, Finn Hallböök, Göran Andersson, Björn Ekesten, Tomas F. Bergström

**Affiliations:** 1 Department of Animal Breeding and Genetics, Swedish University of Agricultural Sciences (SLU), Box 7023, SE-750 07 Uppsala, Sweden; suvi.makelainen@slu.se (S.M.); anna.darlene.heiden@slu.se (A.D.v.d.H.); goran.andersson@slu.se (G.A.); 2 Department of Neuroscience, Uppsala University, Box 593, SE-751 24 Uppsala, Sweden; minas.hellsand@neuro.uu.se (M.H.); finn.hallbook@neuro.uu.se (F.H.); 3 Section of Pathology, Department of Biomedical Sciences and Veterinary Public Health, Faculty of Veterinary Medicine and Animal Science, Swedish University of Agricultural Sciences (SLU), Box 7028, SE-750 07 Uppsala, Sweden; elina.andersson@slu.se (E.A.); elina.thorsson@slu.se (E.T.); 4 Department of Clinical Sciences, Swedish University of Agricultural Sciences, Box 7054, SE-750 07 Uppsala, Sweden; Bodil.Strom-Holst@slu.se (B.S.H.); jens.haggstrom@slu.se (J.H.); ingrid.ljungvall@slu.se (I.L.); bjorn.ekesten@slu.se (B.E.); 5 Canine Genetics Research Group, Kennel Club Genetics Centre, Animal Health Trust, Lanwades Park, Kentford, Newmarket, CB8 7UU Suffolk, UK; cathrynmellersh@yahoo.co.uk

**Keywords:** Bardet–Biedl syndrome (BBS), primary cilia, ciliopathy, BBS8, progressive retinal atrophy (PRA), retinitis pigmentosa

## Abstract

In golden retriever dogs, a 1 bp deletion in the canine *TTC8* gene has been shown to cause progressive retinal atrophy (PRA), the canine equivalent of retinitis pigmentosa. In humans, *TTC8* is also implicated in Bardet–Biedl syndrome (BBS). To investigate if the affected dogs only exhibit a non-syndromic PRA or develop a syndromic ciliopathy similar to human BBS, we recruited 10 affected dogs to the study. The progression of PRA for two of the dogs was followed for 2 years, and a rigorous clinical characterization allowed a careful comparison with primary and secondary characteristics of human BBS. In addition to PRA, the dogs showed a spectrum of clinical and morphological signs similar to primary and secondary characteristics of human BBS patients, such as obesity, renal anomalies, sperm defects, and anosmia. We used Oxford Nanopore long-read cDNA sequencing to characterize retinal full-length *TTC8* transcripts in affected and non-affected dogs, the results of which suggest that three isoforms are transcribed in the retina, and the 1 bp deletion is a loss-of-function mutation, resulting in a canine form of Bardet–Biedl syndrome with heterogeneous clinical signs.

## 1. Introduction

Inherited retinal degenerations (IRDs) are a diverse group of retinopathies leading to visual impairment and blindness in humans and other species. Most of the IRDs, such as retinitis pigmentosa (RP) in humans (OMIM # 268000) and the canine equivalent, termed progressive retinal atrophy (PRA), are non-syndromic and only affect vision. Syndromic IRDs are less common and besides visual impairment, other organs are also affected. In golden retriever (GR) dogs with PRA, a 1 bp deletion in exon 7 of the Tetratricopeptide repeat domain 8 (*TTC8*) gene was identified in 2014 (CanFam3.1 Chr8:60,090,185delA, rs852355138, OMIA 001984-9615, here denoted as *TTC8*^delA^) [1]. This form of PRA is generally referred to as GR-PRA2. The deletion was predicted to cause a frameshift of the open reading frame leading to a premature stop codon in exon 8, 15 codons downstream of the deletion. If translated, the truncated protein would lack most of the tetratricopeptide repeat motifs. In humans, mutations in the *TTC8* gene cause Bardet–Biedl syndrome (BBS; OMIM # 615985), a clinically and genetically heterogeneous autosomal recessive ciliopathy and the second most common human syndromic IRD after Usher syndrome [2,3].

BBS was first described by Georges Bardet and Artur Biedl in the early 1920s [4,5]. The symptoms are highly variable, even between patients from the same family, and are divided into primary and secondary characteristics [6,7]. Primary symptoms include retinal degeneration, obesity, polydactyly, kidney abnormalities, learning disabilities or cognitive impairment, hypogonadism in males, and genital abnormalities in females. Secondary features include speech delay, developmental delay, behavioral abnormalities, eye abnormalities, brachydactyly/syndactyly, ataxia/poor coordination/imbalance, short stature, mild hypertonia, diabetes mellitus, orodental abnormalities, cardiovascular anomalies, situs inversus, hepatic involvement, craniofacial dysmorphism, Hirschsprung disease, and anosmia [2,7,8]. For clinical diagnosis of BBS, it has been suggested that four of the primary characteristics or alternatively three primary and two secondary characteristics should be observed [8].

With 24 genes associated with BBS (OMIM # 209900) to date [9], the syndrome shows large non-allelic heterogeneity [10,11,12,13]. The most common cause of the disorder in humans are mutations in the *BBS1* and *BBS10* genes, each accounting for approximately 20% of human cases [14,15,16]. *TTC8* mutations account for approximately 2% of the cases, being amongst the less frequent causes for BBS [17]. In 2003, Ansley et al. discovered that the *TTC8* gene is associated with BBS and identified the syndrome as a basal body dysfunction of the ciliated cells [2]. The *TTC8* gene, also referred to as *BBS8*, is one of the eight *BBS* genes (*BBS1*, *BBS2*, *BBS4*, *BBS5*, *BBS7*, *TTC8*, *BBS9*, and *BBIP1*), encoding proteins that assemble into a stable octameric protein complex termed the BBSome [18,19]. The BBSome forms a membrane coat that sorts membrane receptors to the primary cilium and its dysfunction leads to the failure of cell-specific signal transduction affecting multiple cell-types and organs [20].

In addition to pleiotropic disorder, there are also reports of non-syndromic retinal degeneration caused by mutations in the BBS genes *BBS1*, *BBS2*, *ARL6*/*BBS3*, and *TTC8* [21,22,23,24], disrupting the normal function of photoreceptor cilia. At the time of the discovery of the canine *TTC8* deletion in 2014, there were indications that dogs homozygous for the deletion might exhibit clinical signs other than retinal degeneration [1]. However, only ophthalmic examinations of the affected dogs were performed and other BBS-associated characteristics could not be clinically investigated. In addition, it was not possible to analyze tissues from affected individuals. Here, we describe a detailed examination of two golden retrievers, homozygous for the *TTC8* variant (*TTC8*^delA^) that were followed from the time of the PRA-diagnosis until they were euthanized and a full necropsy was performed. This allowed for a rigorous clinical characterization of this canine form of a *TTC8*-mediated disease, and to investigate the effect of the canine *TTC8* mutation on the transcriptional and protein level in the canine retina.

## 2. Materials and Methods

### 2.1. Animals and Samples

A golden retriever sib-pair, a male (GR01) and a female (GR02), was followed from the time of their PRA-diagnosis until euthanasia, after which a necropsy was conducted and tissue samples were collected. The male dog was 6 years and 1 month old, and the female was 3 months older at the time of euthanization. Both dogs tested homozygous for the *TTC8*^delA^ allele. Tissue samples were also collected from five unaffected dogs (RW01, BE02, LR02, GS01, and GSP01) euthanized for reasons unrelated to this study (Table S1). In addition, two unaffected dogs (BE01 and LR01; Table S1) were included in the clinical ophthalmic examination (see below). Interviews were conducted with owners of the sib-pair, as well as the owners of eight additional affected golden retrievers, all tested homozygous for the *TTC8*^delA^ allele (Table S2). All samples were obtained with informed consent from the dog owners. Ethical approval was granted by the regional animal ethics committee (Uppsala ethics committee on animal experiments/Uppsala djurförsöksetiska nämnd; Dnr C12/15, Dnr 5.8.18-15533/2018, and Dnr 5.8.18-04682/2020).

### 2.2. Ophthalmic Examination, Confocal Scanning Ophthalmoscopy (cSLO), Optical Coherence Tomography (OCT) and Electroretinography (ERG)

Ophthalmic examination of GR01 and GR02, as well as an unaffected beagle (BE01) and an unaffected Labrador retriever (LR01) included reflex testing, testing of vision with falling cotton balls under dim and daylight conditions, and tonometry (Tonovet, Icare Finland Oy, Vantaa, Finland), as well as indirect ophthalmoscopy (Heine 500, Heine Optotechnik GmbH, Herrsching, Germany) and slit-lamp biomicroscopy (Kowa SL-17, Kowa Company Ltd., Tokyo, Japan) after dilation of pupils with tropicamide (Mydriacyl 0.5%, Alcon Nordic AS, København, Denmark).

cSLO- and OCT-imaging (Spectralis HRT + OCT, Heidelberg Engineering, Heidelberg, Germany) were performed after dilation of pupils with tropicamide and under light sedation with 5 µg/kg medetomidine (Sedator, Dechra Veterinary Products AB, Upplands Väsby, Sweden) and 50 µg/kg butorphanol (Dolorex, Intervet AB, Stockholm, Sweden). The cornea was kept moist using artificial tears throughout the procedure. Total retinal thickness in GR02 was measured as previously described [25], and compared to data from an unaffected 5-year-old female Labrador retriever (LR01) and a 7-year-old female beagle (BE01).

We recorded a bilateral, full-field ERG in GR02 under general anesthesia and compared to data from the unaffected LR01. Sedation with intramuscular acepromazine 0.03 mg/kg (Plegicil vet., Pharmaxim Sweden AB, Helsingborg, Sweden) was followed by induction with propofol 10 mg/kg, intravenously (Propovet, Orion Pharma Animal Health AB, Danderyd, Sweden). After intubation, inhalation anesthesia was maintained with isoflurane (Isoflo vet., Orion Pharma Animal Health AB). Corneal electrodes (ERG-JET, Cephalon A/S, Aalborg, Denmark) were used with isotonic eye drops (Comfort Shield, i.com medical GmbH, Munich, Germany) as a coupling agent. Gold-plated, cutaneous electrodes served as ground and reference electrodes (Grass, Natus Neurology Inc. Middleton, WI, USA) at the vertex and approximately 3 cm caudal to the lateral canthi, respectively. Light stimulation, calibration of lights, and processing of signals were performed as previously described [26] and the ECVO protocol for canine clinical ERGs was followed [27].

### 2.3. Clinical Andrological Examination and Semen Analysis

Semen from the affected male (GR01, age 6 years and 1 month), was collected using digital manipulation with a bitch in estrus present. The sperm concentration was measured using a Bürker chamber. Sperm morphology was evaluated using standard procedures in wet preparations of semen fixed in buffered formalin and in air-dried smears stained with carbolfuchsin–eosin. Due to a very low sperm concentration and volume, only 50 spermatozoa were examined in the wet preparation, under a phase-contrast microscope at 1000× magnification. All abnormalities on any given spermatozoon were counted and the overall frequencies were classified according to a system developed by Bane (1961) [28]. For a more detailed examination of the sperm heads, 100 spermatozoa were evaluated in a smear under a light microscope at 1000× magnification. Presence of spermatogenic cells was recorded in smears stained in accordance with standard well-established methodology. The head morphologies were classified as pear-shaped, narrow at the base, abnormal contour, undeveloped, loose and abnormal, narrow, and variable size.

### 2.4. Echocardiography and Electrocardiographic (ECG) Examinations

For the echocardiographic examinations of the sib-pair (GR01 and GR02, at an age of 6 years and 1 month), the dogs were placed in right and then left lateral recumbency on an ultrasound examination table. The echocardiographic evaluation was conducted by use of an ultrasonographic unit (EPIQ 7G, Philips Ultrasound, Bothell, WA, USA) equipped with a 5-1 matrix transducer and ECG monitoring. The heart was examined and subjectively assessed in standard right- and left-sided views [29]. Blood flow over heart valves was interrogated using color mode Doppler echocardiography and measured using spectral Doppler echocardiography. Left ventricular dimensions were measured using M-mode echocardiography in the right parasternal short axis view at the level of the papillary muscles. The left atrial diameter was measured in the right parasternal short axis view at the level of the aortic valve. Left ventricular dimensions were compared to published weight-based normal reference ranges [30] and left atrial diameter was indexed to the aortic diameter as previously described [31].

### 2.5. Histopathological Examinations

Tissue samples from the affected siblings GR01 and GR02 (Table S3), as well as a retinal sample from an unaffected 3-year-old Rottweiler (RW01), were fixed in 10% neutral buffered formalin for >24 h, paraffin wax embedded, sectioned at 4 µm and stained with hematoxylin and eosin (HE). In addition, kidney sections were also stained with periodic acid-Schiff (PAS). All sections were evaluated using light microscopy.

### 2.6. Total RNA Extraction

The retinal tissue sample from the affected female GR02 was collected directly in TRIzol Reagent (Invitrogen™, Carlsbad, CA, USA) for immediate RNA extraction. Retinal samples from three unaffected dogs—a beagle (BE02), a Labrador retriever (LR02), and a German shepherd (GS01)—were preserved in RNAlater (SigmaAldrich, Saint Louis, MO, USA) directly after euthanasia. The samples were then washed with 1 × PBS and between 50 to 100 mg of tissue was used for extraction. The samples from GR02, BE02, and GS01 were then homogenized in TRIzol Reagent with Precellys homogenizer (Bertin Instruments, Montigny-le-Bretonneux, France) and total RNA was extracted following the manufacturer’s instructions (Pub. No. MAN0001271, Rev. B.0.). Total RNA from LR02 was extracted with RNeasy mini kit (Qiagen, Hilden, Germany) according to the manufacturer’s instructions. RNA concentration was measured using Qubit RNA BR Assay kit (Invitrogen™, Waltham, MA, USA). RNA integrity and quality were inspected with Agilent TapeStation 4150 with an Agilent RNA ScreenTape (Agilent Technologies, Santa Clara, CA, USA). PolyA-selection was carried out utilizing Dynabeads mRNA Purification Kit (Invitrogen™, Waltham, MA, USA), applying the manufacturer-provided protocol.

### 2.7. Long-Read cDNA Sequencing with Oxford Nanopore Sequencing Technology (ONT)

Oxford Nanopore long-read cDNA sequencing libraries for the polyA-selected RNA of the canine retina from the affected GR02 as well as unaffected BE02 and GS01 were prepared using the Direct cDNA Sequencing kit (SQK-DCS109, Oxford Nanopore Technologies Ltd., Oxford, UK) following the manufacturer’s instructions (Protocol version DCS_9090_v109_revC_04Feb2019; updated 02 May 2019). The input material for each library was 100 ng of polyA-selected RNA. The prepared libraries were sequenced on a MinION sequencer using R9.4.1 flow-cells and MinKNOW (v.19.06.7) software. The resulting raw fast5 files were basecalled using Guppy version 3.1.5+781ed57 with the --trim_strategy DNA flag. The quality control for each run was performed using an R markdown script developed by ONT (Nanopore_SumStatQC_Tutorial.Rmd). To characterize the retinal *TTC8* transcripts, quality passed reads (quality score > 7) were mapped to the canine reference genome sequence CanFam3.1 using MiniMap2 (v.2.16) with -ax splice parameter (Li, 2018) [32]. A reference-guided assembly of the transcriptome was produced using StringTie2 (v.2.1.1) with default settings for long-reads (-L) [33], with Ensembl anotation build 100 (CanFam3.1). The assembled transcripts were compared to the raw sequnce data using The Integrative Genomics Viewer (IGV) [34,35]. Transcripts with less then 20× coverage were discared. To quantify the expression levels, quality passed reads (quality score > 7) were mapped to the canine reference transcriptome consisting of protein coding gene sequences and non-coding RNA sequences from Ensembl build 100 (CanFam3.1) using Minimap2 (v2.16) with the settings -ax map-ont -N 100 [32]. The transcript counts were produced using Salmon (v.1.1.0) in aligned based mode and --libType A for automatic detection of library type [36]. The results for each transcript expressed as transcripts per million (TPM) were summarized to produce gene level TPM values. We then analyzed the expression levels of genes considered to be expressed in a cell-specific manner (retinal marker genes) [37,38,39,40,41,42,43,44,45,46,47,48,49,50,51,52,53,54,55,56,57]. The complete list of marker genes and their respective references can be found in Table S4.

### 2.8. Quantitative RT-qPCR

cDNA was synthesized using RNA prepared from retinal samples of the affected female (GR02) and three unaffected dogs (BE02, LR02, and GS01) using RT^2^ First Strand kit (Qiagen, Hilden, Germany) with random hexamers provided in the kit. cDNA concentration was measured using Qubit ssDNA Assay kit (Invitrogen™, Waltham, MA, USA). To amplify the target and the reference genes, custom primers were designed using the software Primer3 [58,59] (Table S5). The cDNA fragments encoding regions of interest were amplified using RT^2^ SYBR Green ROX qPCR Mastermix (Qiagen, Hilden, Germany) on a StepOnePlus Real-Time PCR system (Applied Biosystems™, Waltham, MA, USA), according to the manufacturer’s instructions. Target gene expression was normalized to expression of *GAPDH* as well as *ACTB* reference genes, and shown relative to a control sample (BE02) (△△CT method). The results were confirmed in two independent experiments.

### 2.9. Fluorescence Histochemistry

Retina from the affected female (GR02) and an unaffected 10-year-old male German spaniel (GSP01) were analyzed by means of fluorescence histochemistry. The lens and anterior segment were removed and the vitreous punctured before the eye cups were fixed in 4% PFA in 1 × PBS for 15 min on ice, washed in 1 × PBS for 10 min on ice, and cryoprotected in 30% sucrose in 1 × PBS at 4 °C until saturated. The eye cups were embedded in Neg-50™ frozen section medium (Thermo Scientific, Waltham, MA, USA) and 10 µm sections were collected on Superfrost Plus slides (J1800AMNZ, Menzel-Gläser, Thermo Fisher Scientific, Waltham, MA, USA). Sections were re-hydrated in 1 × PBS for 10 min, incubated in blocking solution (1% donkey serum, 0.02% thimerosal, and 0.1% Triton X-100 in 1 × PBS) for 30 min at room temperature, and incubated in blocking solution containing Alexa Fluor™ 488-conjugated PNA (1:400, L21409, Invitrogen™, Waltham, MA, USA) and a rhodopsin primary antibody (1:5000, NBP2-25160, Novus Biologicals, Abingdon, UK) overnight at 4 °C. The sections were then washed in 1 × PBS, 3 × 5 min, incubated in Alexa 568 secondary antibody (1:2000, A10037, Invitrogen™, Waltham, MA, USA) for at least 2 h at room temperature, washed in 1 × PBS, 3 × 5 min, and the slides mounted using ProLong™ Gold Antifade Mountant with DAPI (P36931, Molecular Probes, Waltham, MA, USA). Images were captured using a Zeiss Imager.Z2 microscope equipped with an Axiocam 512 mono camera (Carl Zeiss Microscopy GmbH, Jena, Germany).

## 3. Results

### 3.1. Desciption of Primary and Secondary Bardet-Biedl Syndrome Characteristics

To investigate if the dogs exhibit other BBS-related problems, we interviewed the owners of 10 affected dogs. Two of the dogs were also part of the 2014 study [1], and we based the interviews on the questionnaire developed by Downs et al. (2014) [1]. Among the 10 affected dogs, 4 were reported to exhibit a minimum of four characteristics matching human primary BBS-signs (Table S2). The remaining dogs displayed three (two dogs) and two (three dogs) primary signs, respectively, and for one dog, PRA was the only reported primary characteristic. All of the dogs had been diagnosed with PRA, and the average age of diagnosis was 4 years and 8 months. None of the dogs were reported to have polydactyly or any other malformations of the paws, but obesity or polyphagia, renal problems, cognitive dysfunction, irregular estrous cycles, and decreased libido in males were among the reported primary signs. As for secondary BBS signs, many dogs were described to have a short stature relative to the standard for the breed. Partial or complete loss of the sense of smell (anosmia), worsening with age, was noticed by most of the owners (Table S2). Half of the individuals were also diagnosed with cataracts. At the time of this study, seven of these dogs were no longer alive. The oldest dog was reported to have died in his sleep at 10 years of age, while the other dogs had been euthanized at an average age of 7.5 years. Among the dogs in the questionnaires was the BBS-affected sib-pair (GR01 and GR02), which were further investigated in this study.

### 3.2. Ophthalmic Examinations of the Affected Sib-Pair

The male dog GR01 was initially diagnosed with PRA at the age of 4 years and 3 months, after which he and his female littermate GR02 were genotyped and found to be homozygous for the *TTC8*^delA^ allele and subsequently recruited for this study. The two dogs were then examined ophthalmologically on three occasions: at the age of 4.5 and 5.5 years, and finally at the age of 6 years and 1 month. At the first examination, both dogs had mildly dilated pupils in room light. Pupillary light reflexes and menace responses were considered normal, but the dazzle reflex was mildly impaired in both eyes (oculi uterque, OU). The results of the cotton ball test in room light were unremarkable for both dogs, whereas the female detected approximately 50% and the male only between 10–20% of the cotton balls under dim light conditions. On indirect ophthalmoscopy, generalized abnormal tapetal reflection going from hypo- via normo- to hyperreflection (in the male) was seen when the indirect ophthalmoscopy lens was tilted back and forth. Furthermore, retinal vascular attenuation and pigment clumping in the non-tapetal fundus were observed. Compared to the female dog, the male showed signs of more advanced retinal degeneration. Findings were symmetrical between eyes. In addition to the abnormal retinal appearance, equatorial and anterior cortical cataracts were seen in the eyes of both individuals. The male had multiple, pigmented iridociliary cysts mainly emerging through the nasal portion of the pupils (OU), but free-floating and collapsed cysts were also seen in the anterior chambers. Intraocular pressures (IOP) were normal (OU) in both dogs.

**Figure 1:**
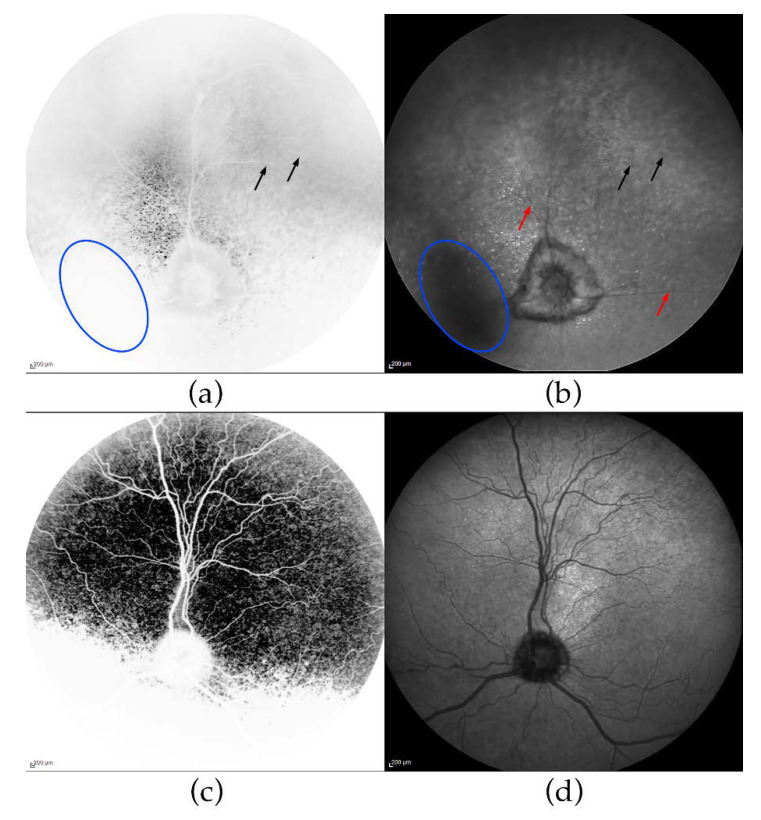
Fundus autofluorescence (FAF) and infrared confocal scanning laser ophthalmoscopy (IR-cSLO). cSLO FAF- and IR-cSLOs of the left eye of GR02 (panels (**a**,**b**), respectively) and age-matched, unaffected Labrador retriever LR01 (panels (**c**,**d**), respectively). Both FAF-images are inverted and hence, the tapetal reflection in GR02 (**a**) is remarkably faint. Detection of FAF in the tapetal fundus is difficult because of the normal, bright reflection from the tapetum lucidum. Black arrows indicate areas with bright reflection in the tapetal fundus on FAF (**a**), but hyporeflection on IR (**b**), which is suggestive of lipofuscin accumulation. The slight hyperreflection (the grey shade below the optic nerve head in the inverted image) (**a**) in the non-tapetal area autofluorescence indicates storage of lipofuscin. The IR-image (**b**) shows the large, myelinated optic nerve head often seen in the golden retriever breed, but very attenuated retinal vessels (red arrows) and islets of hyperreflectivity. Cast shadows in the cSLOs from GR02 were caused by the cataracts present in this dog (one shadowed area indicated by the blue oval).

To further investigate the retinal phenotype, we performed cSLO and OCT on the affected female (GR02). Reduced fundus autofluorescence (FAF) in the entire tapetal fundus was seen on cSLO (Figure 1a), whereas the non-tapetal fundus appeared slightly hyperreflective indicating accumulation of lipofuscin in the degenerating retina. Retinal vascular attenuation in the affected fundus was evident, as well as islets with bright tapetal hyperreflection (Figure 1b), compared to the unaffected dog (Figure 1c,d).

**Figure 2:**
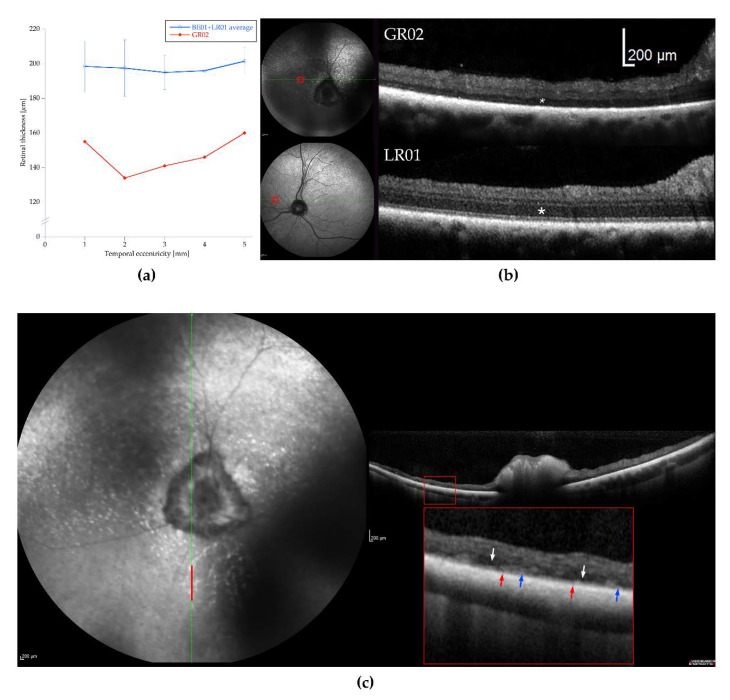
Retinal thickness and optical coherence tomography (OCT). (**a**) Total retinal thickness temporally from the optic nerve head (ONH) towards the periphery of the retina is reduced in the affected dog (GR02) compared to the thickness observed in an unaffected Labrador retriever (LR01) and a Beagle (BE01). (**b**) OCTs from the nasal part of the fundus (red rectangles) from the affected dog (GR02, top) and the unaffected dog (LR01, bottom) showing both the marked thinning of the outer nuclear layer (ONL) (asterisk) and less distinct segmentation of retinal layers in the affected dog. (**c**) Appearance of the retina in the non-tapetal, ventral fundus of GR02. The vertical red bar in the cSLO-image shows the area outlined by the red rectangle on the OCT photograph, which is then magnified below (the area inside the large red rectangle). Cast shadows on the cSLO-image were caused by cataracts. The thinned retina is more variable in thickness than in the tapetal fundus dorsal to the ONH. The retinal pigment epithelium (RPE) is occasionally fragmented (red arrows) or appears thickened (blue arrows). The segmentation of the outer retina is difficult and dark areas probably representing clusters of nuclei in the ONL are seen occasionally (white arrows).

OCT showed considerable retinal thinning (Figure 2a) compared to two unaffected dogs (LR01 and BE01). Mainly the outer retinal layers were reduced in thickness and outer retinal landmarks, such as the external limiting membrane (ELM), ellipsoid zone (EZ), and interdigitation zone (IZ), could not easily be identified in the affected dog (Figure 2b). The inner nuclear layer (INL) was thin and often fragmented, making the segmentation of the outer plexiform layer (OPL) and inner plexiform layer (IPL) difficult. In the ventral (inferior) non-tapetal fundus, retinal thickness was also reduced, but more irregularly, giving a patchier appearance. Again, segmentation of the outer retinal layer on the OCT image was difficult and a distinct outer nuclear layer ONL occasionally missing. Both fragmentation and thickening of the retinal pigment epithelium (RPE)was observed (Figure 2c). The cataracts in the male (GR01) hindered the cSLO and OCT examinations.

**Figure 3:**
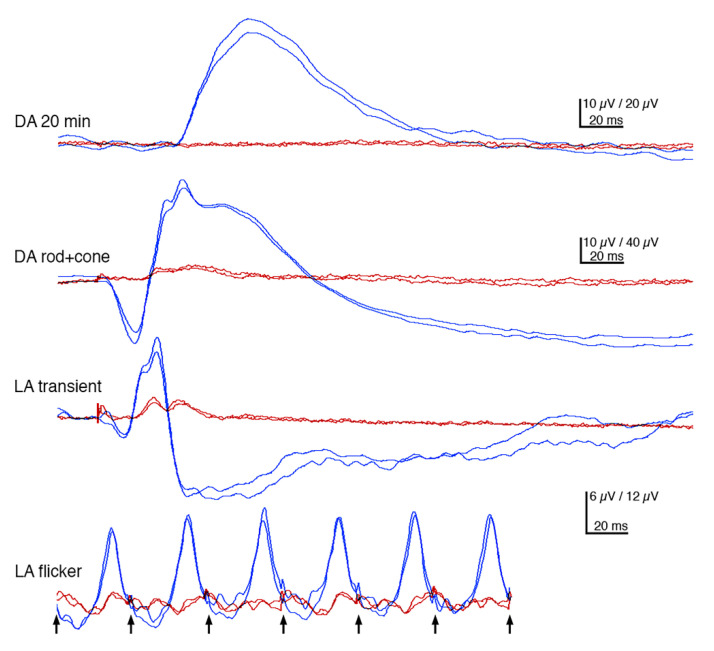
Flash-electroretinographs (FERGs). Both dark-adapted (DA) and light-adapted (LA) responses of the FERG had subnormal amplitudes in the affected golden retriever (GR02; red tracings) compared to the unaffected Labrador retriever (LR01) (blue tracings). Rod responses were essentially non-detectable and even the dark-adapted responses to the bright rod-cone stimulus seem to be mainly cone-driven. Light-adapted cone transients lacked a-wave and had a biphasic waveform. When a 30 Hz stimulus was employed, the cone responses of GR02 came out of sync and were not time-locked to the individual stimuli. Calibrations show the time base and the different amplitude scales for GR02 and LR01.

Flash-electroretinography (FERG) was performed in the female, but not in the male, because of the ophthalmoscopic signs of more advanced retinal degeneration. In the female, rod responses were non-detectable throughout the 20 min dark adaptation. Neither was the a-wave of the mixed, dark-adapted rod-cone response discernible and the b-wave had profoundly subnormal amplitude and was dominated by the early, mainly cone-driven part. The light-adapted cone-driven transient b-wave was relatively better preserved, but biphasic with a late, second peak, whereas the a-wave was essentially missing. The cone flicker response was more abnormal, as the cone-driven responses were unable to follow the 30 Hz stimuli (Figure 3).

The siblings were re-examined 11 months later at the age of 5.5 years. Pupils were now moderately to widely dilated in both dogs, pupillary light reflexes sluggish and incomplete, and dazzle reflexes impaired. The male dog showed poor menace responses and did not respond to falling cotton balls regardless of lighting, but did occasionally follow hand movements at 30 cm in bright light. Vision was less impaired in the female; menace responses were retained and approximately 50% of the falling cotton balls were perceived in room light, whereas no cotton balls were detected in dim light. Ophthalmoscopy showed generalized tapetal hyperreflection in both dogs with a brighter hyperreflection in the male than in the female. Retinal vascular attenuation was now moderate to advanced and the optic nerve heads appeared pale. In the non-tapetal area, pigment clumping was observed, as on the previous examination. Cataractous changes had progressed in both dogs and were seen in both the anterior and posterior cortices of both eyes, as well as in the equatorial region. Iridociliary cysts were still present in the male and his eyes were normotensive.

**Figure 4:**
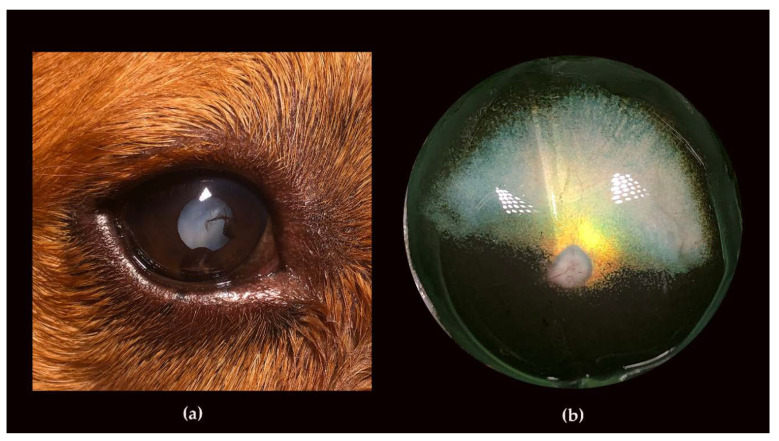
Iridociliary cysts and fundus imaging of the affected male (GR01). (**a**) Iridociliary cysts both at the posterior side of the iris and free-floating in the anterior chamber were seen in both eyes of the affected male (GR01). Pigment and remnants of ruptured cysts were seen on the anterior capsules of the cataractous lenses. (**b**) The eyecup of the affected male (GR01), *postmortem.* Bright tapetal hyperreflection is seen close to the optic nerve head (ONH), whereas the rest of the tapetum is faintly colored. The retinal vessels, including the larger venules, are almost impossible to follow.

Both dogs were examined a third time at the age of 6 years and 1 month. At this time, the male was severely visually impaired both under daylight and dim light conditions, and had developed bilateral mature-hypermature total cataracts. In addition, the dog showed both intact and ruptured iridociliary cysts bilaterally (Figure 4a) and had normal IOP. However, the ocular fundi could not be examined in vivo because of the cataracts. Postmortem gross examination of the fundi revealed bright hyperreflection in the tapetal area (Figure 4b). The network of retinal blood vessels was severely attenuated (even for a postmortem specimen) and difficult to follow towards the periphery (Figure 4b). The pupils of the female dog (GR02) were dilated in room light conditions, the pupillary light reflexes (PLRs) were sluggish and incomplete, dazzle reflexes were poor, and menace responses were present in room light, but not in dim light conditions. The female was unable to detect falling cotton balls regardless of lighting. The cataractous changes had progressed (OU), particularly in the posterior than in the anterior cortex, and the tapetal hyperreflectivity and retinal vascular attenuation could now only be seen like through a haze.

### 3.3. Andrological Examination

**Figure 5:**
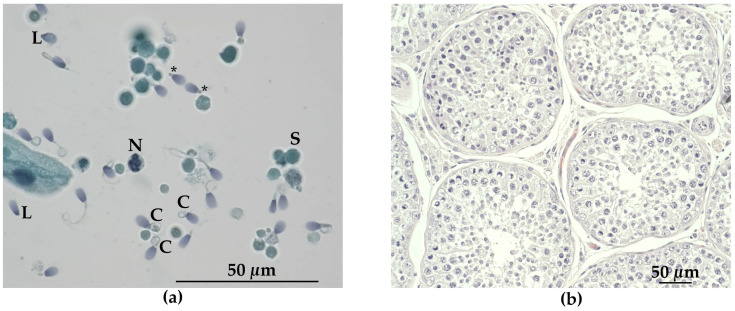
Light microscopy of sperm and seminiferous tubules from the affected male (GR01). (**a**) Papanicolaou staining of semen showing several abnormal spermatozoa. Different tail defects are evident, including coiled tails (C), tail stumps (*) and loose heads (L). S: spermatogenic cell, N: neutrophil leukocyte. (**b**) Tubuli seminiferi in testis stained with hematoxylin and eosin, showing mainly early stages of spermatogenesis and few elongated spermatids (ES).

The testes of the affected male dog GR01 were both palpable in the scrotum and found to be smaller than normal for the age and breed. An ejaculate of a very small volume (<0.5 mL), and light grey in color, was obtained. The sperm concentration was 15 × 10^6^/mL and motility was <5%. The total sperm count could not be calculated exactly, but was estimated to be <7 × 10^6^/mL. There was a high proportion of spermatozoa with abnormalities, and less than 1% were considered morphologically normal. Midpiece defects were predominant (82% of spermatozoa), consisting of “Dag defect” [60], with strong folding, coiling, and fracture of the distal part of the midpiece, and “tail stump defect”, where, in place of normal tails, short “stumps” were found. Other defects were coiled tails (12%), and double bent tails (6%), as well as proximal droplets (12%), acrosome defects (4%), and nuclear pouches (20%). Head defects were detected in 36% of the spermatozoa, the most common being pear shaped (11%), variable size (10%), and loose abnormal heads (7%). In addition, a large number of spermatogenic cells, inflammatory cells, and epithelial cells, as well as necrotic cells of varying sizes, were present (Figure 5a).

### 3.4. Cardiac Examination

Both dogs (GR01 and GR02) presented normal respiratory sinus arrhythmia at a normal heart rate (between 80–90 BPM). Echocardiography showed normal cardiac morphology, dimensions, and motion in both dogs. Minimal regurgitation over the mitral valve was detected in both dogs, but valve morphology appeared normal. None of the dogs manifested *situs inversus*.

### 3.5. Necropsy Findings

To further investigate clinical features associated with BBS, a thorough necropsy was conducted on both affected dogs. The body condition score (BCS) of the male (GR01) was estimated to be 9/9, consistent with obesity [61]. In gross examination of the male, an abnormal head shape with a broadened muzzle and flat forehead was perceived, as was a low withers height. Furthermore, there was gingival hyperplasia and moderately increased interdental spaces (diastema) in both lower and upper jaw. The testicles were also seemingly small for the dog’s breed and age. Gross examination of the kidneys revealed suspected chronic infarcts bilaterally, with the most marked changes in the right kidney (Table S3). Mild myxomatous valvular degeneration was seen in the heart of the affected male. The body condition score (BCS) of the female (GR02) was assessed to be 7/9, consistent with heavy [61]. The interdental spaces were also moderately increased. The kidneys showed signs of mild chronic glomerulonephritis bilaterally (Table S3).

### 3.6. Histopathology Findings

Next, we examined the histopathology of tissue samples collected during the necropsy. Abnormal changes were observed in the retina, kidney, and testis. The majority of the retina from the male (GR01) exhibited severe retinal thinning with complete loss of normal architecture with most pronounced degenerative changes in the non-tapetal fundus. Compared to a normal retina (Figure S1a), the ONL of the affected male GR01 was severely affected in the tapetal fundus and completely missing in the non-tapetal fundus (Figure S1b,c).

**Figure 6:**
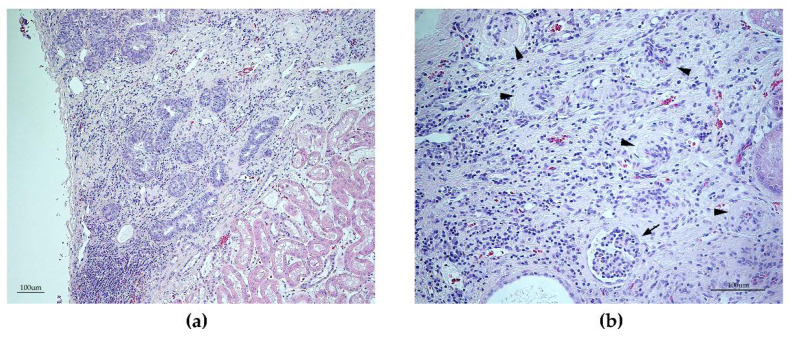
Renal histopathology of the affected male dog (GR01). Light microscopy of the right kidney stained with hematoxylin and eosin, showing a segmental area of fibrosis, (**a**) infiltrated by lymphocytes and plasma cells, extending from cortex, with the presence of a disarray of atypical tubular structures, and (**b**) with occasional small glomeruli with peripheral nuclei and inapparent capillaries (fetal glomeruli, marked with an arrow). Furthermore, multiple degenerated glomeruli are seen (arrowheads).

Histopathological examination of the testicles revealed only few (or no) late spermatids in the tubuli seminiferi (Figure 5b). A reduced number of spermatocytes was also observed. Microscopically, sections from the lesions in the right kidney of the male (GR01) corresponded to segmental areas of fibrosis, infiltrated by a moderate number of lymphocytes and plasma cells, extending from cortex to medulla (Figure 6a,b). In the fibrotic tissue, there were some degenerated glomeruli and lack of tubular structures. Furthermore, a disarray of occasional small glomeruli with peripheral nuclei and inapparent capillaries (fetal glomeruli), as well as atypical tubular structures outlined by pseudostratified cuboidal epithelium with large, basophilic, plump cells, were seen. No cellular atypia or mitotic activity were evident. Occasional dilated tubular structures were seen (Figure 6b). No chronic infarcts were confirmed histologically in the left kidney. On microscopic examination of the kidneys from the female (GR02), changes consistent with mild chronic glomerulonephritis was evident.

### 3.7. Characterization of Canine TTC8 Transcripts in the Retina

To comprehensively characterize the different *TTC8* transcripts expressed in the canine retina and to investigate the effect of the 1 bp deletion (*TTC8*^delA^) in an affected dog, we performed full-length cDNA sequencing of the neural retina from the female (GR02), as well as of two 12-year-old unaffected female dogs, a beagle (BE02) and a German shepherd (GS01), using Oxford Nanopore Technologies (ONT) for long-read sequencing. This produced 3.6 M reads and 5.86 Gb of quality passed DNA sequence for GR02, 7.8 M reads and 10.68 Gb for BE02, as well as 7.6 M reads and 7.06 Gb for GS01 (Table 1).

**Figure 7:**
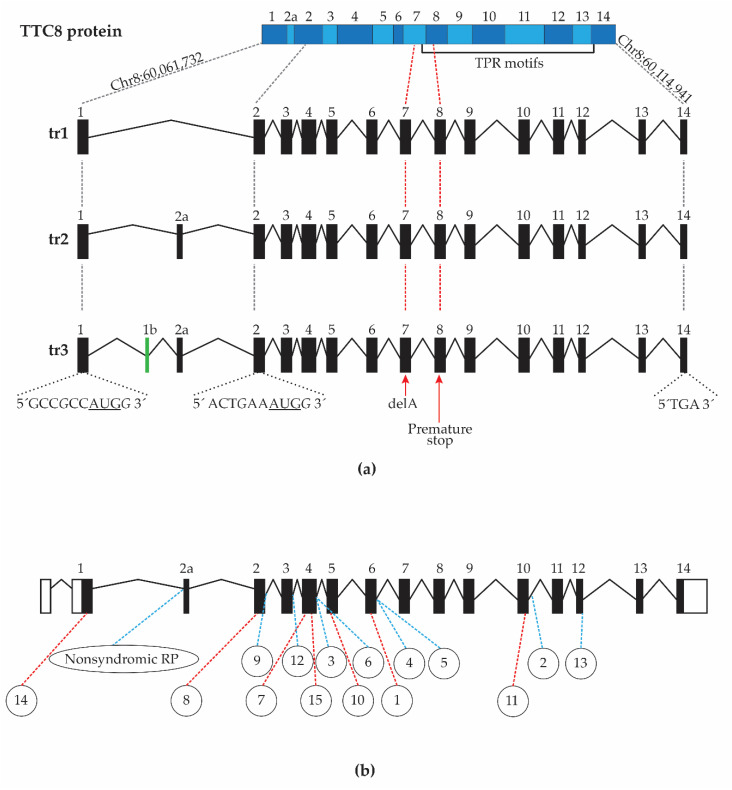
Retinal isoforms of canine the *TTC8* gene and human variants associated with Bardet-Biedl syndrome (BBS). (**a**) On top, a schematic representation of the TTC8 protein including the approximate location of the TPR motifs, and below, the three identified canine retinal transcripts. The black dashed lines indicate the two alternative TIS including the Kozak consensus sequence, and the translation termination site in exon 14. The red dashed lines indicate the 1 bp deletion of adenine in exon 7 and the premature stop codon in exon 8. (**b**) An overview of identified human *TTC8* coding variants (red dashed line) and splice-site variants (blue dashed line), see Table S6. The positions of the human mutations are based on the reported positions in the original publications, and the exon numbering may therefore differ from the positions in the presented transcript (ENST00000345383.9). tr1: transcript 1, tr2: transcript 2, tr3: transcript 3, TPR: tetratricopeptide repeat, TIS: translation initiation site.

The cDNA sequence data was assembled into transcripts using reference-guided assembly. The predicted transcripts of the *TTC8* gene were then manually curated with the raw sequence data. We could not assemble any full-length *TTC8* transcripts from the sample of the affected dog (GR02) due to lack of read coverage over the *TTC8* locus. In contrast, sequence data from the unaffected dogs (BE02 and GS01) suggested transcription of three different retinal isoforms at the *TTC8* gene, here denoted tr1, tr2, and tr3 (Figure 7a). Fourteen exons were detected in tr1, 15 in tr2, and 16 in tr3, comprising 6.5%, 76.1%, and 17.4% of the *TTC8* transcripts, respectively. In tr1, exons 1b and the retinal specific exon 2a were missing. In tr2, exon 1b was absent, whereas tr3 featured both exons 1b and 2a. The comparison of the identified transcripts to existing annotations showed that the exon-intron boundaries and sequence between the first and the last exon of tr1 corresponds to the curated NCBI RefSeq transcript NM_001284469.1, tr2 corresponds to the predicted NCBI RefSeq transcript XM_003639207.4, as well as Ensembl transcript ENSCAFT00000027700.5, and tr3 corresponds to the predicted NCBI RefSeq transcript XM_014115661.2. The exon-intron boundaries of the identified transcripts show similarity to human *TTC8* transcripts (Figure 7b). In all three transcripts, a putative translation initiation site (TIS) was identified in exon 1 (Chr8:60,061,732) containing an AUG start codon with an adjacent optimal (GCCRCCAUGG) Kozak consensus sequence (Figure 7a) [62,63]. Using the TIS, tr1 and tr2 have an open reading frame extending to the last exon, encoding for 505 and 515 aa protein products, respectively. However, in tr3, the TIS would lead to a termination codon two exons downstream of exon 1, producing a 49 aa protein product and would lack the tetratricopeptide repeat (TPR) motifs. An alternative TIS is found at position Chr8:60,077,223 of exon 2 and, although the adjacent Kozak consensus is not optimal, it is classified as strong (NNNRNNAUGG) [63]. This alternative TIS is in-frame with the original coding sequence, and its open reading frame would produce a 455 aa protein product for all three transcripts, including the TPR motifs (Figure 7a). We did not observe any transcripts skipping exon 7, where the deletion Chr8:60,090,185^delA^ is located, nor any exons downstream the deletion.

**Figure 8:**
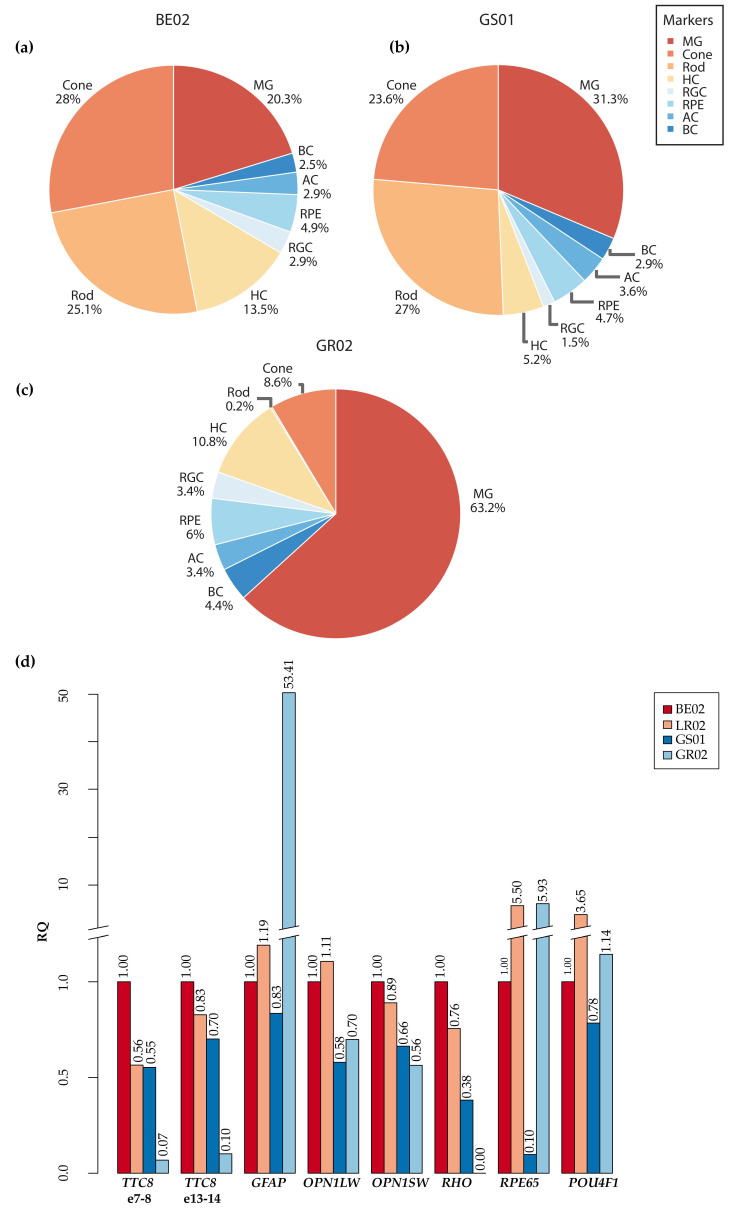
Expression of retinal marker genes. (**a**) The average expression of marker genes for each retinal cell type in the unaffected BE02 (**a**) and GS01 (**b**) dogs as well as the affected GR02 (**c**), based on cDNA sequencing. (**d**) Relative mRNA expression levels by quantitative RT-qPCR in two different regions (exons 7–8 and exons 13–14) of the *TTC8* gene, as well as the retinal marker genes *GFAP* (macroglial cells), *OPN1LW* and *OPN1SW* (cone photoreceptors), *RHO* (rod photoreceptors), *RPE65* (RPE cells), and *POU4F1* (retinal ganglion cells) expression in three unaffected dogs (BE02, LR02, GS01) and the affected GR02, normalized to *GAPDH* and *ACTB* gene expression. Rod: rod photoreceptors, cone: cone photoreceptors, RGC: retinal ganglion cells, AC: amacrine cells, HC: horizontal cells, BC: bipolar cells, MG: macroglial cells, RPE: retinal pigment epithelium, BE02: unaffected beagle, GS01: unaffected German shepherd, GR02: affected golden retriever, LR02: unaffected Labrador retriever.

Next, we compared *TTC8* gene expression levels of the unaffected BE02 and GS01 with the *TTC8* levels of the affected GR02 with 1 bp deletion in the exon 7 (Figure 8). We first estimated the transcript abundance for each annotated gene with Salmon (v.1.1.0) [36] using canine transcriptome from Ensembl build 100 and then summarized the level of transcript expression of each gene in the annotation. In BE02, the *TTC8* gene was expressed at a level of 99.8 transcripts per million (TPM) (856th most highly expressed gene), and in GS01 at a level of 60.6 TPM (859th most highly expressed gene). As suggested by the low read coverage of GR02 of *TTC8* transcripts, the expression levels were low in the affected dog, with 16.8 TPM (3625th most highly expressed gene). We then summarized the expression levels of known marker genes for different cell types in the retina (Table S4, Figure 8). The expression of genes transcribed in the rod photoreceptor cells (*PDE6A*, *PDE6B*, *CNGB1*, *CNGA1*, and *GNAT1*) [39,43,64,65,66] accounted for 25–27% of the total marker gene expression (TPM) in both the unaffected dogs (Figure 8a,b). For the affected GR02, the expression was estimated to 0.2%, indicating that the sample only included a small fraction of rod cells compared to the unaffected dogs (Figure 8c). The rhodopsin gene (*RHO*), encoding for a specialized G protein-coupled receptor known to be expressed exclusively in rod photoreceptors, was not included in the most recent Ensembl annotation release (build 100) used in the analysis, and therefore not included in the rod marker gene list. Similarly, the POU Class 4 Homeobox 1 (*POU4F1*), marker for retinal ganglion cells [67], was not among the genes annotated in Ensembl build 100. Both *RHO* and *POU4F1* were instead included in the quantification using reverse transcription quantitative real-time PCR (see below). The expression levels of cone photoreceptor cell markers (*ARR3*, *GUCA1C*, *PDE6C*, *PDE6H*, and *OPN1LW*) [37,38,39,40,41,42,43] were also lower in GR02, whereas the expression levels of macroglial cell (MG) marker genes (*RLBP1*, *SLC1A3*, *GLUL*, *CLU*, and *GFAP*) [43,44,45,46] were elevated (Figure 8a–c). The marker gene expression levels of the horizontal cells (HC) [47,48], retinal ganglion cells (RGC) [49,50,51,52,53,54], RPE cells [50,55], amacrine cells (AC) [47,56], or bipolar cells (BC) [57] did not differ drastically between the three dogs, although the proportional expression of BC and RPE markers was higher in the affected female. The complete list of marker genes and their expression values in each sample can be found in Table S4.

To verify the relative differences in the expression levels of *TTC8* and retinal marker genes in the affected GR02 and unaffected BE02 and GS01, as well as in an unaffected Labrador retriever (LR02, not used in the cDNA sequencing), we performed reverse transcription quantitative real-time PCR (RT-qPCR). We amplified two separate regions of the *TTC8* gene over the exons 7 to 8 and 13 to 14 (Figure 8d). In addition, we amplified canine long-wave (*OPN1LW)*, and short-wave (*OPN1SW*) opsins expressed in cone photoreceptors, as well as, retinoid isomerohydrolase RPE65 (*RPE65*) and glial fibrillary acidic protein (*GFAP*) genes (markers for RPE cells and macroglia, respectively) to compare their cDNA sequencing levels to RT-qPCR. We also used *RHO* to evaluate relative rod photoreceptor levels and POU Class 4 Homeobox 1 (*POU4F1*) to evaluate retinal ganglion cells in these retinal samples. With the exception of *RPE65* expression, the RT-qPCR results reflected the cDNA sequencing quantification showing that the expression of both *TTC8* amplicons, as well as *RHO* expression, were lower in the GR02 compared to the three unaffected dogs and *GFAP* expression was higher than in the unaffected dogs (Figure 8d).

### 3.8. Fluorescence Histochemical Analysis of the TTC8^delA^ Retina

**Figure 9:**
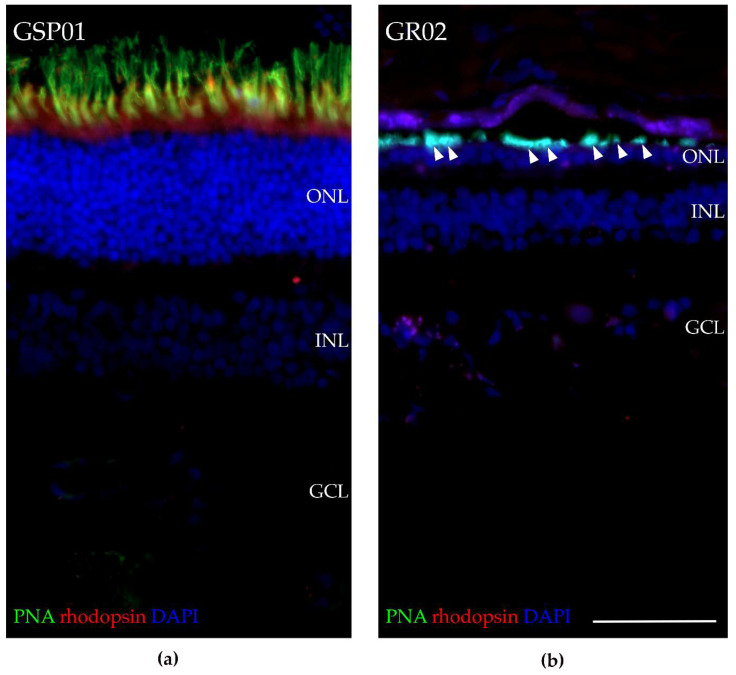
Fluorescence histochemistry of dog retinas for rhodopsin and cone photoreceptors. Fluorescence micrograph showing rhodopsin expression (red), Alexa™ 488-conjugated PNA (green), and DAPI (blue) in (**a**) a male, unaffected, 10-year-old German spaniel (GSP01) and (**b**) a female, affected, 6-year-old golden retriever (GR02). Note the thin ONL, lack of rhodopsin staining, and the truncated cone outer segments (exemplified with arrowheads) in the retina of the affected compared that of the unaffected dog. PNA: peanut agglutinin, ONL: outer nuclear layer, INL: inner nuclear layer, GCL: ganglion cell layer. Scale bar: 50 µm and applies to both images.

RT-qPCR analysis revealed low expression of markers of rod and, to a lesser extent, cone photoreceptors in the affected female (GR02; Figure 8), and the histopathology analysis for the affected male (GR01; Figure S1) and OCT analysis for the affected female (GR02; Figure 2) showed that both the ONL and photoreceptor layer were thinner compared to control retinas. To investigate the presence of photoreceptors in the *TTC8*^delA^ retina, we performed fluorescence histochemistry using an antibody directed against rhodopsin and fluorophore-conjugated peanut agglutinin (PNA), which binds selectively to cone photoreceptors in the retina [68]. Retinal sections from an unaffected, 10-year-old German spaniel (GSP01) and the affected, 6-year-old female golden retriever (GR02) were analyzed (Figure 9). We found that the ONL and the photoreceptor layer were thinner in the GR02 retina compared to control. In the unaffected retina, both rhodopsin and PNA staining were found in the photoreceptor layer (Figure 9a), whilst in the affected GR02 retina, rhodopsin staining was absent and PNA labeled what appeared to be truncated cone outer segments in the photoreceptor layer (Figure 9b). The fluorescence data corroborate the histopathology and OCT findings.

## 4. Discussion

When the deletion at the *TTC8* locus (*TTC8*^delA^) in golden retrievers was discovered in 2014, there were indications that the mutation did not only lead to progressive retinal degeneration in the affected dogs, but could also cause additional clinical signs, suggesting a syndromic type of disease [1]. However, at the time, no cases were available for a thorough clinical examination apart from ophthalmoscopy, and no tissue samples could be collected. With the increasing number of genetically affected dogs detected by DNA-testing, we were able to continue the questionnaires and interviews initiated by Downs et al. The results strengthened the view that the disease in golden retrievers (generally referred to as GR_PRA2) may indeed be similar to Bardet–Biedl syndrome (BBS) in humans. To diagnose BBS patients correctly is often challenging due to the heterogeneity of the disease with symptoms that vary even between individuals within families carrying identical mutations. In addition, the symptoms overlap with other diseases, such as Laurence–Moon syndrome, which may in fact, to some extent be considered a variation of the same condition [69]. Polydactyly or similar congenital digit abnormalities can be cues in the search for a correct diagnosis. In the absence of digit malformations, the relatively early onset of retinal degeneration [6,7], can guide clinicians towards a BBS diagnosis. Both polydactyly and retinal degeneration are primary characteristics of BBS, as well as obesity, kidney abnormalities, cognitive impairment, hypogonadism in males, and genital abnormalities in females [7]. To date, 15 mutations in the human *TTC8* gene have been reported to cause BBS [2,17,70,71,72,73,74,75,76] (Table S6; Figure 7b). These mutations include six intronic splice-site variants, as well as three exonic splice-site variants (one missense, one nonsense, and one silent mutation). In addition, the identified mutations include three frameshift insertions/deletions, one complex variant, one nonsense variant, and one non-frameshift deletion in the coding sequence. Moreover, one *TTC8* splice-site variant has been associated with non-syndromic RP in humans [24]. Interestingly, one of the exonic splice-site variants (NM_144596.3: c.1347G>C; p.Gln449His) has been reported to cause both non-syndromic RP [77] and BBS [70], suggesting that the genetic background of each patient may play a significant role in the development of BBS symptoms in humans. Similar to humans, the genetic background is also likely to result in the heterogeneous clinical signs in dogs (Table S2).

All the dogs in this study were diagnosed with PRA initially causing visual problems in dim light, later also under daylight conditions and eventually causing blindness. This is consistent with BBS, where rod-cone dystrophy is the most frequently observed clinical sign, diagnosed in about 93% of the patients with approximately three out of four becoming legally blind by the second to third decade of life [6,7]. FERGs from the middle-aged female dog (GR02) showed bilateral loss of rod function, as well as profoundly subnormal and delayed cone-amplitudes, supporting a diffuse rod-cone degeneration (Figure 3). This is similar to human patients with *TTC8* mutations, where loss of rod and cone function has been reported [24,72,77]. Early in the course of the disease, human BBS patients may have cone flicker responses with marked delays and near normal amplitudes that deteriorate over time [78]. *Ttc8*-knockout mouse models show both reduced rod and cone ERGs at an early age [79]. Interestingly, the mouse models also demonstrate that cone structure, function, and viability depend on the normal expression of *Ttc8.* Loss of the TTC8 protein results in shortened and disorganized photoreceptor outer segments already before retinal maturation, as early as post-natal day 10 in *Ttc8*-knockout mice [79]. The lack of rhodopsin staining, and the appearance of truncated cone outer segments observed in the retina of the affected female using fluorescence histochemistry (Figure 9), suggests that the deletion at the *TTC8* gene (*TTC8*^delA^) eventually results in a photoreceptor phenotype similar to the *Ttc8*-knockout mice. Taken together, the progressive reduction of cone photoreceptors and concomitant decline of cone ERGs may not only be secondary to degeneration of rods, as the TTC8 protein appears to be essential for normal cone function.

We were able to investigate the retina of the affected female using OCT, and considerable outer retinal thinning was observed (Figure 2), corroborating the results from ERG and postmortem findings. The abnormal appearance and thinning of the INL was judged as secondary to the photoreceptor degeneration. OCT-scans from the ventral (inferior) retina showed a more patchy degeneration with irregular RPE lining, also reported in some human patients with BBS [80], and clusters of nuclei in the ONL. Accumulation of lipofuscin, most clearly seen as increased autofluorescence in the non-tapetal fundus (Figure 1) has also been reported in human patients with BBS. However, the precise distribution of lipofuscin in the canine fundus is difficult to determine on cSLO because of the tapetal reflection in this species.

In addition to retinal degeneration, both the clinically investigated siblings, as well as three other affected dogs, were diagnosed with cataracts, a common secondary characteristic in human BBS [78]. In contrast to the posterior polar cataracts typically seen in human patients, both the affected male and the female developed cataracts that rather rapidly spread in the posterior, equatorial, and anterior cortices. The cataracts contributed to the dogs’ visual impairment. The prevalence of posterior polar cataracts in human BBS has been reported to be similar in patients with different genotypes, although none of the patients studied was homozygous for pathogenic mutations in the *TTC8* gene [81]. We cannot exclude that the cataracts of the affected dogs were secondary to the retinal degeneration, because secondary cataracts are frequently seen in late-stage hereditary photoreceptor degenerations (PRA) in dogs [82]. Toxic dialdehydes from decaying photoreceptors have been proposed to induce cataract formation [83] and the location of the cataractous changes in GR01 and GR02 in the more metabolically active equatorial and cortical regions of the lenses may also suggest a toxic effect on the lens. However, the cataracts in the golden retriever sib-pair were observed already at the initial examination when the degeneration of the retinae was judged as moderately advanced.

The affected male was also diagnosed with iridociliary cysts (Figure 4a). Iridociliary cysts have previously been reported in golden retrievers with glaucoma [84] and retinal detachments, but have not been associated with progressive retinal degenerations. Our observation of iridociliary cysts in the affected male is most likely a coincidental finding, but could possibly be a result of the deletion at the *TTC8* gene and a consequence of abnormal ciliary trafficking.

When Ansley et al. identified *TTC8* as a novel BBS gene in 2003 [2], situs inversus was observed in one of the patients with a *TTC8* splice-site mutation. This led to the important conclusion that BBS is a disease caused by ciliary dysfunction. The TTC8 protein is part of the BBSome, an octameric protein complex which functions in the exit of activated G protein–coupled receptors (GPCRs) from cilia [19,85,86]. Thus, the underlying cause of the syndrome is malfunction of primary cilia, an organelle emanating from the cell surface of most mammalian cell types during growth arrest [87]. Primary cilia function in cell signaling during development and in homeostasis, thus explaining the multitude of organs affected in each BBS patient and the repertoire of different symptoms experienced. Nodal cilia, a type of motile primary cilia transiently present during embryonal development, are crucial for breaking the left-right symmetry during the embryogenesis, and defects in this process may cause different forms of heterotaxy such as partial or total situs inversus, as well as congenital heart defects [88,89]. Manifestation of situs inversus and other forms of heterotaxy is rare in the general population (1/10,000). However, in a study of 368 participants in the Clinical Registry Investigating Bardet–Biedl Syndrome (CRIBBS), six patients (1.6%) were found to have disorders of asymmetry, suggesting a 170-fold increase compared to the general population [90]. Cardiovascular diseases and congenital heart defects have also been reported for human BBS patients [7,91]. Of the 19 patients with *TTC8* mutations (Table S6), 3 were diagnosed with hypertension or haemophilia [2,72,76]. We therefore investigated the cardiac status of the two affected golden retrievers. Both dogs showed normal cardiac morphology, dimensions and motion, and a normal left-right body axis. In the postmortem gross examination, a mild myxomatous valvular degeneration was seen in the heart tissue of the affected male. While the finding is common in dogs [92], it has recently been shown that defects of the primary cilia in the extra-cellular matrix can lead to myxomatous mitral valve disease in humans and mice [93].

Hypogonadism in males and genital abnormalities in females are considered primary characteristics of human BBS [6,7]. Hypogonadism was also common for the male patients with *TTC8* mutations (Table S6). The affected the males had a low total sperm count and a high number of abnormal sperm cells, mainly lacking flagellum or having other tail-defects, indicating infertility (Figure 5a). He did not display any interest in the female dog in heat at the time of sampling. The histopathological examination of the testes revealed only a few elongated spermatids (Figure 5b). Severe sperm defects, including tail abnormalities as well as a low total sperm count, have previously been reported in Hungarian Puli dogs with a loss-of-function mutation in the Bardet–Biedl syndrome 4 (*BBS4*) gene [94]. The importance of BBS-genes for the normal formation of flagella in spermatogenesis has further been shown in mouse models. Genetically modified mice for any of the genes *Bbs1* [95], *Bbs2* [96], *Bbs3* (*Arl6*) [97], *Bbs4* [98], *Bbs6* (*Mkks)* [99], or *Bbs7* [100], result in failure to form normal flagella. In a *Bbs8*-knockout mouse model, the sperm defects were not described, but the males were found to be infertile [79].

Interestingly, many of these mouse models, including the *Ttc8* knockout mouse [101], also showed partial or complete anosmia. This is a secondary characteristic experienced by many BBS patients in general [102], but it was not reported for the 19 human patients with *TTC8* mutations. This is in contrast with the present study, where most of the owners of the affected dogs (8/10) reported their dogs having poor or gradually worsening sense of smell (Table S2). This appear to be a characteristic that is shared between dogs and mice, but not with humans. Moreover, as was the case for the dogs, none of the mouse models exhibited polydactyly, and the phenotype of dogs may therefore resemble the mouse phenotype more closely than that of human patients with *TTC8* mutations.

Obesity was reported for 16 of the 19 human patients. Although we did not formally investigate the body condition score for more than two dogs, the majority of them appeared overweight or heavy according to the owners (Table S2). It should, however, be noted that we have not compared this to the general golden retriever population. Similar to the two golden retriever siblings in this study, dental anomalies were reported for one human patient with a *TTC8* splice-site mutation [76] and for 27% of the BBS patients in general [7].

The histopathological changes in the right kidney of the affected male were most similar to a renal dysplasia-like lesion (Figure 6). The female sibling had mild chronic bilateral glomerulonephritis, but such mild chronic inflammatory changes are an unspecific finding, which probably did not affect the animal clinically. In total, 4 of the 10 dogs included in this study had renal problems (Table S2), suggesting that, as in humans, mutations in the *TTC8* gene may result in variable renal phenotypes. Among the 10 golden retrievers included in this study (Table S2), 1 dog was reported to be euthanized due to kidney failure. Other reasons for euthanasia included gastrointestinal problems (2 dogs), as well as problems related to sensory deprivation (visual and olfactory impairments: 2 dogs) and neoplastic disease (1 dog). Interviews indicated a rather low average lifespan of the affected golden retrievers: 7 years and 8 months for the nine dogs in the questionnaires (one of the ten dogs is still alive as of preparing this manuscript, 5 years of age). Data from 1995–2002 show that almost 90% of golden retrievers survive to 8 years of age and more than 80% to the age of 10 [103]. The 1 bp deletion in the canine *TTC8* gene had detrimental effects on the dogs’ health and longevity. In human BBS patients, kidney failure is a frequent cause of death and the average survival of the patients overall is substantially reduced [104]. Taken together, the deletion in the canine *TTC8* gene is associated with additional clinical features apart from PRA in the affected dogs, such as obesity, renal and genital anomalies, anosmia, short stature, and dental anomalies that are similar to human BBS.

The major isoforms of the TTC8 protein are predicted to consist of approximately 500 amino acid residues, and the exon-intron structure is well conserved between human, dog, and mouse. Two canine retinal transcripts were previously predicted to exist based on Sanger sequencing of cDNA by Downs et al (2014) [1], one with and one without the retinal specific exon termed 2a. The transcript with the retinal specific exon was originally reported in humans [24]. It was shown that an in-frame splice-site mutation leading to the skipping of exon 2a is sufficient to cause a non-syndromic retinal degeneration in humans. In mice, the retinal specific exon 2a was found to be exclusively expressed in the ocular tissue, having the highest expression in the outer segments (OS) of both rod and cone photoreceptor cells [24,105]. In addition to retinal photoreceptor cells, exon 2a has also been found expressed in pinealocytes of rat pineal gland, and it has been suggested that these two cell types have evolved from a common precursor photodetector cell [106]. In our study, we used Oxford Nanopore Sequencing Technology for long-read sequencing, capable of reading through full-length transcripts and therefore enhancing the detection of different splice sites. Notably, the data only gave support for three different transcripts expressed in the retinal tissue (Figure 7), while the Ensembl annotation (release 100) predicts the existence of seven canine transcripts, of which only one (ENSCAFT00000027700.5 corresponding to tr2) was identified in this study. The six additional Ensembl canine transcripts are either skipping exon(s): E5 (ENSCAFT00000043691.2), E6 (ENSCAFT00000050179.3), E5-6 (ENSCAFT00000093101.1), E2a-10 (ENSCAFT00000070341.1), E1-2a (ENSCAFT00000086744.1), or E1-5 (ENSCAFT00000087482.1). We found no evidence for the presence of these other transcripts in our data from the canine retina.

As expected, the tr2 transcript, which includes the retinal specific exon 2a, was found to be the most highly expressed of the three identified transcripts. We also identified a short transcript (tr1) without the retinal specific exon 2a, with low expression in the dog retina. In mice, despite having a lower expression than the retinal specific transcript, the short transcript lacking the exon 2a is expressed throughout the retinal cell layers and shows the highest expression in the RPE [24]. The retinal samples investigated in this study did not exclusively include photoreceptor cells, but also consisted of other neural cells and RPE cells (Figure 8), which likely express the short transcript tr1. In addition to these two previously identified canine retinal transcripts, we also found evidence for a third transcript (tr3) including both exon 2a as well as a short exon with 22 nucleotides, here denoted 1b. A transcript similar to tr3 is also reported in GenBank (XM_014115661.2), likely annotated by an ab initio prediction detecting an open reading frame of sufficient length, but likely without supporting alignment (The NCBI Eukaryotic Genome Annotation Pipeline; A translation initiation site (TIS) with an optimal Kozak consensus sequence was identified in exon 1, but in tr3 this would extend an ORF of 147 nucleotides over the exons 1b and 2a, and reach a stop codon in exon 2 (Figure 7). This short transcript would likely be degraded by nonsense-mediated decay (NMD) [107]. If an alternative TIS was used in exon 2, tr3 would be in-frame with the other transcripts and produce a protein product of 455 amino acid residues featuring the complete TPR motif, which is involved in protein-protein interaction [108]. It is yet to be defined if this transcript is translated into a protein product, and if its expression is tissue specific.

The expression levels for all three *TTC8* transcripts found in this study were markedly reduced in the affected female dog. This was seen in the quantification of the cDNA sequencing data (Figure 8a–c), but also by the lack of read coverage of transcripts mapping to the *TTC8* locus in the dog reference genome sequence when manually inspecting the data in IGV. The highest read depth in the locus was 13× (TPM = 16.8), while the read coverage for the two unaffected dogs (250–500×, TPM = 60.6 and 99.8, respectively) was clearly higher than in the affected female. The reads detected in the affected dog appeared to be only partly spliced and none of the reads reached full-length over all exons. The low levels of *TTC8* transcripts in the affected dog was further supported by RT-qPCR (Figure 8d). The data suggested that the deletion in exon 7 (*TTC8*^delA^) and the subsequent premature stop codon in exon 8 lead to degradation of the *TTC8* transcripts in the affected individual. To estimate the cell type constitution of the samples, we also studied the expression levels of retinal marker genes in the affected female compared to the two unaffected female dogs. As suggested by the histological sectioning of the affected male retina (Figure S1) and the fluorescence histochemistry of the affected female (Figure 9), the sequencing results showed a drastically lower expression of rod photoreceptor cell-specific genes, and cone photoreceptor cell marker expression was only one third compared to the levels detected in the two other dogs (Figure 8a–c, Table S4). The same pattern was observed in the RT-qPCR, where we quantified the relative expression of *RHO* and *OPN1LW* genes (Figure 8d). The retinal degeneration of both of these dogs (GR01 and GR02) was therefore likely advanced. BBSome deficiency has been shown to cause defects in the transport of phototransduction proteins between the inner and outer segments of the photoreceptors and, ultimately, these defects lead to photoreceptor cell death [96,109]. In addition to aberrant photoreceptor marker gene expression, our data also suggested that the expression of macroglial genes (Müller glia and astrocytes) was higher in the affected dog (Figure 8), most notably clusterin (*CLU*) and glial fibrillary acidic protein (*GFAP*) expression (Table S4), both of which are known to be upregulated under retinal stress and retinal degeneration [110,111,112,113].

## 5. Conclusions

The *TTC8* gene encodes for one of the proteins forming the BBSome, and has in humans been implicated in Bardet–Biedl syndrome (BBS). Long-read cDNA sequencing of non-affected dogs suggested the expression of three retinal *TTC8* transcripts and that the 1 bp deletion is a loss-of-function mutation. Golden retriever dogs homozygous for the deletion develop an autosomal recessive form of RP-like retinal degeneration (PRA), but it has hitherto been unclear if the affected dogs develop a non-syndromic PRA or a syndromic ciliopathy similar to human BBS. In addition to PRA, we have shown that the loss-of-function mutation indeed causes additional clinical features, such as obesity, renal and genital anomalies, anosmia, short stature, and dental anomalies. We therefore conclude that the deletion can result in a canine form of BBS. As in humans, BBS in dogs appear to be a heterogeneous disorder with variable severity of clinical and morphological signs. A canine model for BBS may be of importance for novel therapeutic management of human patients. Canine models have successfully been used to establish protocols for gene therapy of other inherited retinal diseases, and a *TTC8*-dog model could potentially be developed to restore vision and improve the quality of life for BBS patients.

## Figures and Tables

**Table 1:** Full-length cDNA sequencing (ONT).

Dog	Reads Produced	Bases Called *	Quality Passed Reads	Quality Passed Bases *	Mean Read Length (bp)	N50 (bp)	Mean Read Quality
GR02	4,895,849	6.78	3,577,000 (73.1%)	5.86 (86.3%)	1637	2277	10.1
BE02	10,284,735	12.36	7,819,514 (76%)	10.68 (86.4%)	1365	1756	10.6
GS01	11,717,598	9.01	7,629,750 (65.1%)	7.06 (78.4%)	926	1135	9.5

* Gb of DNA sequence.

## Supplementary Materials

The following are available online at https://www.mdpi.com/2073-4425/11/9/1090/s1, Figure S1: Retinal changes associated with *TTC8*^delA^, Table S1: List of dogs included for OCT and/or collection of tissue samples, Table S2: Primary and secondary characteristics, Table S3: Macroscopic and microscopic examination, Table S4: Marker gene expression, Table S5: Primers, Table S6: Human *TTC8* mutations.
