# Supplementary figures and images for: Deletion in the Bardet–Biedl Syndrome Gene TTC8 Results in a Syndromic Retinal Degeneration in Dogs
Source: Genes (Basel). 2020 Sep 18;11(9):1090. doi: 10.3390/genes11091090 (PMC7565673; doi:10.3390/genes11091090)

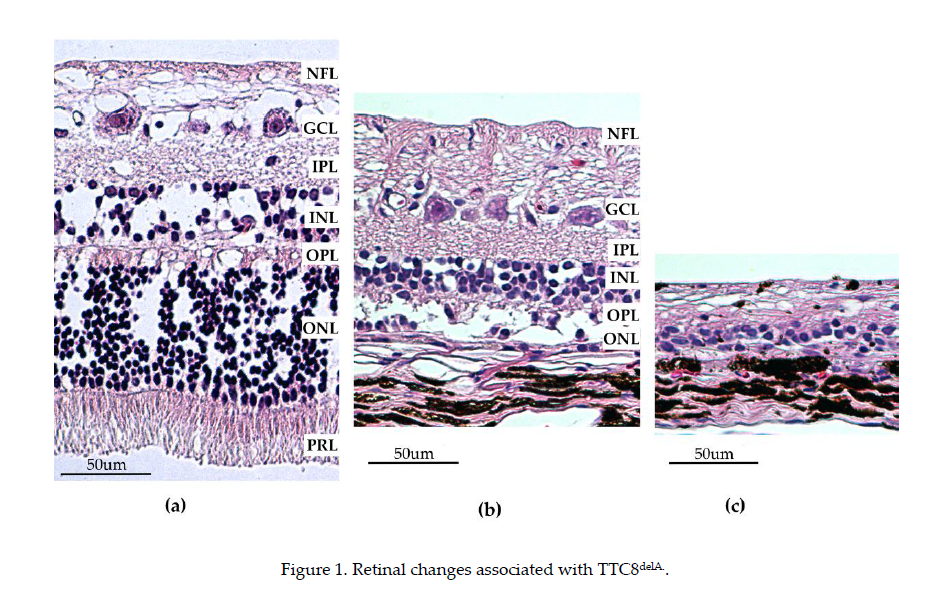

Supplement: Supplementary file 1 [file genes-11-01090-s001.zip › genes-11-01090-s001/genes-926292-supplementary/figure S1.png]
